# Neurological Complications of COVID-19 Infection: A Comprehensive Review

**DOI:** 10.7759/cureus.65192

**Published:** 2024-07-23

**Authors:** Travis Brauer, Sulaiman Paika, Roshni Kotwani, Deepesh Khanna

**Affiliations:** 1 Medicine, Nova Southeastern University Dr. Kiran C. Patel College of Osteopathic Medicine, Clearwater, USA; 2 Foundational Sciences, Nova Southeastern University Dr. Kiran C. Patel College of Osteopathic Medicine, Clearwater, USA

**Keywords:** neurological complications with covid-19, guillain-barré syndrome (gbs), meningoencephalitis, posterior reversible encephalopathy syndrome (pres), miller-fisher syndrome, cerebellar-ataxia, olfactory dysfunction, neurological signs and symptoms, covid-19, sars-cov-2

## Abstract

The COVID-19 pandemic is well on its way to reaching endemic status across the globe. While the medical community’s understanding of the respiratory complications induced by COVID-19 is improving, there is still much to be learned about the neurological manifestations associated with COVID-19 infection.

This review aimed to compile relevant, available evidence of COVID-19-induced neurological complications and to provide information for each complication regarding symptomology, progression patterns, demographic risk factors, treatment, and causative mechanism of action when available.

Data for this review was collected using a confined search on PubMed using the keywords [“COVID-19” OR “SARS-CoV-2”] AND [“neurological complications” OR “olfactory symptoms” OR “gustatory symptoms” OR “myalgia” OR “headache” OR “dizziness” OR “stroke” OR “seizures” OR “meningoencephalitis” OR “cerebellar ataxia” OR “acute myelitis” OR “Guillain Barré Syndrome” OR “Miller Fisher Syndrome” OR “Posterior Reversible Encephalopathy Syndrome”] between 2019 and 2023.

A wide range of neurological manifestations impact a significant percentage of COVID-19 patients, and a deeper understanding of these manifestations is necessary to ensure adequate management. The most common neurological complications identified consist of olfactory and gustatory dysfunctions, myalgia, headache, and dizziness, while the most severe complications include stroke, seizures, meningoencephalitis, Guillain-Barré syndrome, Miller Fisher syndrome, acute myelitis, and posterior reversible encephalopathy syndrome.

While this review effectively provides a roadmap of the neurological risks posed to COVID-19 patients, further research is needed to clarify the precise incidence of these complications and to elucidate the mechanisms responsible for their manifestation.

## Introduction and background

COVID-19 is caused by severe acute respiratory syndrome coronavirus 2 (SARS-CoV-2), which most commonly presents with flu-like symptoms that primarily impact the respiratory system. These symptoms include fever, cough, nasal congestion, and fatigue, and in severe cases, it can progress to acute respiratory distress syndrome (ARDS), characterized by diffuse inflammatory damage to the alveoli-capillary border [1]. However, COVID-19’s clinical manifestations are not limited to the respiratory tract, as seen with other viral infection agents. Gastrointestinal, cardiovascular, and renal manifestations have also been well-documented [2-9]. Gastrointestinal symptoms include diarrhea, vomiting, anorexia, and abdominal pain [10]; cardiovascular complications include arrhythmias, myocarditis, myocardial infarction, and venous thromboembolism [11]; and renal manifestations range from mild proteinuria to acute kidney injury (AKI) requiring renal replacement therapy [12,13]. Throughout the pandemic, the rise of multiple lethal coronavirus variants, coupled with lapses in public health policies, significantly contributed to the global proliferation of the virus [14-18]. As of March 21, 2024, the CDC (Centers for Disease Control and Prevention) COVID Data Tracker reports that 6,880,585 hospitalizations and 1,184,376 deaths have resulted from COVID-19 infection in the United States through March 9, 2024 [19]. Worldwide, the WHO (World Health Organization) COVID-19 dashboard indicates that 774,834,251 cases of COVID-19 infection have been reported, and 7,037,007 deaths from COVID-19 have occurred through March 3, 2024 [20].

A system that has begun to receive increased scrutiny for COVID-19-induced complications is the neurological system [10-13,21]. Many of the most common symptoms of COVID-19 infection, such as anosmia, dysgeusia, myalgia, headache, and dizziness, are attributable to impairment of the neurological system. However, there is also a growing list of less common, more severe neurological complications, including seizures, stroke, meningoencephalitis, cerebellar ataxia, acute myelitis, Guillain-Barré syndrome (GBS), Miller Fisher syndrome (MFS), and posterior reversible encephalopathy syndrome (PRES) [21]. This review aims to compile available evidence of the most relevant COVID-19-induced neurological symptoms and complications and to provide information for each sequela regarding symptomology, incidence, progression patterns, demographic risk factors, treatment, and possible mechanism of action (MOA).

## Review

Materials and methods

**Figure 1:**
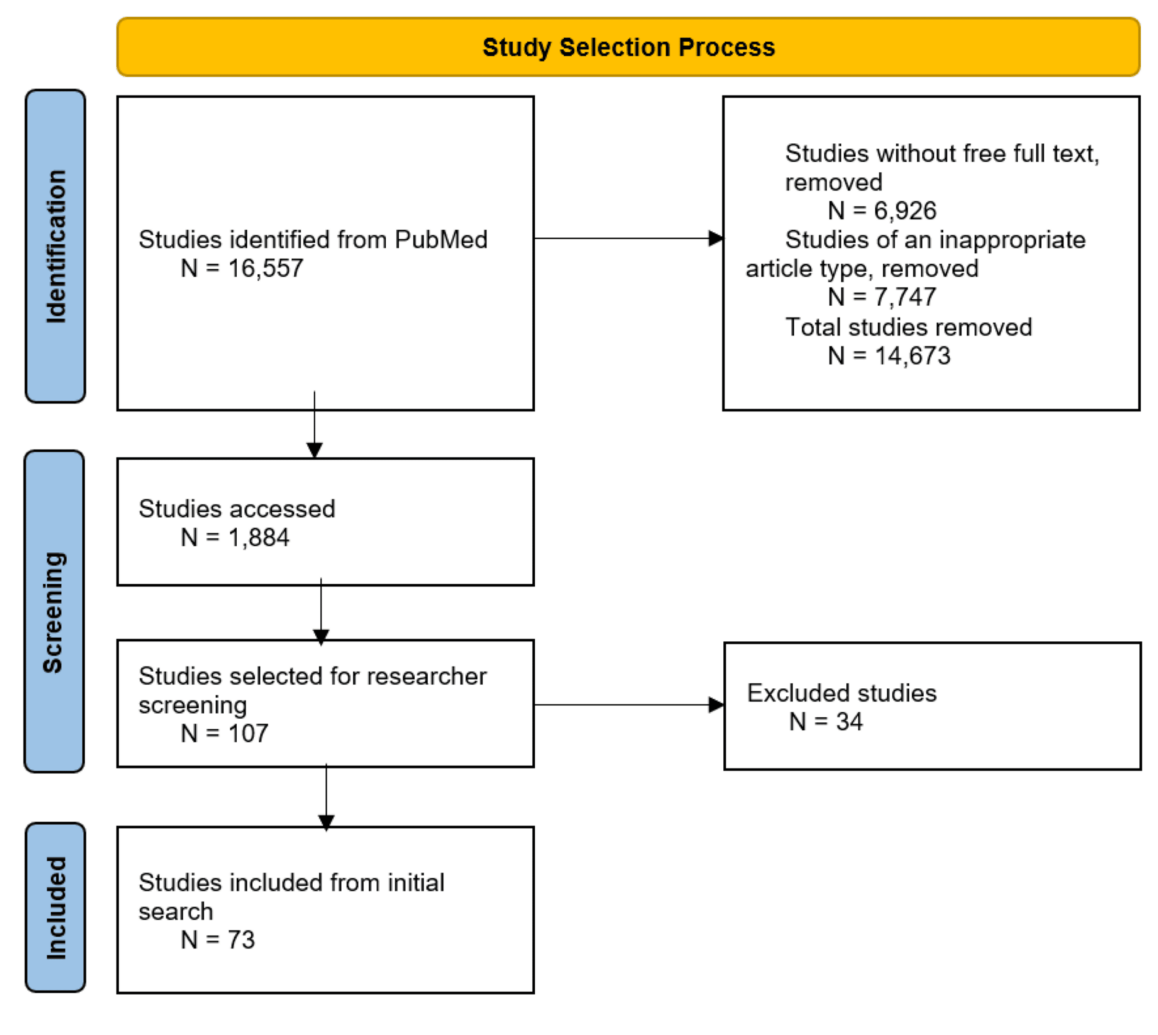
Study selection flowchart

Data for this review were collected using a confined search on PubMed with the keywords [“COVID-19” OR “SARS-CoV-2”] AND [“neurological complications” OR “olfactory symptoms” OR “gustatory symptoms” OR “myalgia” OR “headache” OR “dizziness” OR “stroke” OR “seizures” OR “meningoencephalitis” OR “cerebellar ataxia” OR “acute myelitis” OR “Guillain Barré Syndrome” OR “Miller Fisher Syndrome” OR “Posterior Reversible Encephalopathy Syndrome”] between 2019 and 2023. Articles containing either clinical or mechanistic information concerning neurological sequelae as a result of COVID-19 disease were included, while articles focusing on non-neurological manifestations of COVID-19 disease were excluded. The types of sources selected were primary sources, such as case studies, or knowledge syntheses, such as systematic reviews, meta-analyses, and literature reviews. All included articles from PubMed were peer-reviewed. Figure 1 shows the study selection flowchart.

Discussion

The following section lays out neurological symptoms or complications reported to be induced by COVID-19. For each sequela listed, the authors sought to provide information regarding symptomology, incidence, progression patterns, demographic risk factors, treatment, and MOA whenever available. Table 1 provides a summary of the key findings for each of the sequelae.

**Table 1:** Summary of the key findings for each of the sequelae IVIg: Intravenous immunoglobulin

Sequelae	Symptoms	Incidence	Presentation timeline	Risk factors	Treatment
Olfactory/gustatory dysfunctions	Hyposmia, anosmia, hypogeusia, ageusia [21]	Hyposmia - 15.7%; anosmia - 63.4%; hypogeusia - 68.8% [21]	Early in COVID-19 infection; reported as the earliest symptom of COVID-19 infection in 17.1% of patients [21]	Female predominance, less severe COVID-19 cases [22]	None; sensory rehabilitation for patients 40 and above with both anosmia and ageusia [23]
Myalgia	Generalized muscle pain, aching [24-27]	Ranges from 20% of COVID-positive patients [24] to 66.1% of COVID-positive patients [25]	Early in COVID-19 infection; 66.1% of patients at time of diagnosis [25]	Female predominance [27]	Painkillers [28]
Headache	New onset, bilateral in the temporoparietal, occipital, and forehead regions with a pressure-like pain [29]	37.7% [30]	Present at onset [29]	No prevailing risk factors	Tramadol [31]
Dizziness	General dizziness can present as unsteady walking [32]	16.8% [33]	5-6 days post-infection [34]	Female predominance [35]	NA
Stroke	Unilateral facial droop, dysarthria, unilateral upper extremity weakness, endovascular catastrophe [36]	0.4-1.4% [37]	Inconsistent presentation due to varying pathologies	Those already at risk for stroke [38]	Prophylactic anticoagulation regiment [39]
Seizures	Varying; episodic loss of awareness, "weak legs", and loss of bladder control [40]	0.5% of COVID patients [40]	Inconsistent presentation due to varying pathologies	NA	Keppra (levetiracetam), Ativan (lorazepam), phenytoin, phenobarbital, benzodiazepines [40]
Meningoencephalitis	Seizures, altered mental status, nuchal rigidity, photophobia, nausea, vomiting [41,42]	NA (low incidence)	4-12 days post-infection [43]	Those with latent viral/fungal infections that can cause meningoencephalitis [44]	IVIg [45]
Cerebellar ataxia	Dysarthria, dysmetria, dysdiadochokinesia, truncal ataxia, broad-based gait, difficulty walking [46-53]	NA (low incidence)	0-48 days post-infection [47]	Male predominance [47]	IV glucocorticoids, IVIg if necessary [46,48,49,52,53]
Acute myelitis	Fever, headache, muscle pain, muscle weakness, paresthesia [54,55]	1.43-4.6 cases per million [56]	Onset 7-14 days post-infection is most common [55,56]; concurrent presentation is also possible [54]	2nd to 4th decade of life following infection [54]	IV glucocorticoids; if no response, plasmapheresis or immunomodulatory therapy [57]
Guillain-Barré syndrome	Sensory dysfunctions, paraparesis, tetraparesis, generalized lower limb areflexia, dysphagia, and cranial nerve deficits [58]	NA (low incidence)	Varying timeframes; after non-neuro COVID-19 manifestations - 94.5%; together with non-neuro manifestations - 1.8%; before onset of non-neuro manifestations - 3.6% [59]	Male predominance, less favorable outcomes with increased age [58,59]	IVIg [59,60], plasmapheresis, steroids [59]
Miller Fisher syndrome	Ophthalmoplegia, ataxia, areflexia, dysphagia, dysphonia, diplopia, hypoesthesia, and back pain [58-62]	NA (low incidence)	Onset 3-20 days after non-neuro manifestations [58]	Male predominance, less favorable outcomes with increased age [58,59]	IVIg [58-62]
Posterior reversible encephalopathy syndrome	Headache, altered consciousness/mentation, visual disturbances, seizures, and fluctuations in blood pressure [60,63]	NA (low incidence)	Day 25 [64], day 25, and day 26 [65] after COVID-19 infection, all the following extubation	Severe COVID-19 cases, patients requiring ventilation in the course of COVID-19 treatment [63,65,66]	Manage blood pressure (IV nicardipine to oral nicardipine) followed by physical therapy if necessary [65]

Olfactory/Gustatory Dysfunctions

Olfactory and gustatory dysfunctions have come to be recognized as frequently associated symptoms of COVID-19. Symptoms of hyposmia and anosmia, as well as hypogeusia and ageusia, tend to present suddenly during the onset of COVID-19, as evidenced by a study of 1,043 COVID-19 positive patients, 17.1% of whom experienced a decreased sense of smell as the earliest symptom [21]. Several studies have touched on the persistence of gustatory and olfactory dysfunction symptoms relative to more classic sinonasal symptoms. One study found that out of 3,386 COVID-19 patients, about 60% of the women and about 48% of the men reported less than 80% of their pre-COVID-19 smelling abilities 200 days after onset [67]. In another study of 128 non-hospitalized COVID-19 patients at 229 days post-onset, both olfactory and gustatory dysfunctions persisted in 48% and 38.5% of patients, respectively, with olfactory and gustatory recovery being positively correlated with one another [68]. These symptoms are more commonly found in younger female patients with mild cases of the virus [22]. The main suggested pathogenic mechanism for these symptoms is the high-affinity binding of angiotensin-converting enzyme 2 (ACE2) receptors by the SARS-CoV-2 receptor binding domain (RBD) in the nasal mucosa, as well as in sustentacular cells of the nasopharynx. This high-affinity binding results in cellular dysfunction, anosmia, and ageusia. Additionally, this binding process may disrupt the epithelium and result in an inflammatory response, which similarly contributes to olfactory and gustatory dysfunctions [69]. A more recent study has also found evidence of quantitative olfactory dysfunctions persisting in patients with long COVID-19, including phantosmia (the detection of smell in the absence of an olfactory stimulus) and parosmia (a distorted sense of smell). In this study, 824 patients took an at-home smell test, known as the Odorized Marker Test, and were evaluated for several parameters, including parosmia, which 43.2% of participants noted, and phantosmia, which 36.9% of patients noted. Interestingly, 41.9% of women versus only 26.1% of men reported phantosmia, underlining the potential impact of sex on qualitative olfactory dysfunction [70]. While there are no specific pharmacological treatments proposed for these symptoms, sensory rehabilitation has been suggested for patients aged 40 and above with both anosmia and ageusia [23].

Myalgia

Myalgia has proven to be another of the most common COVID-19 symptoms, with studies ranging in prevalence from 21% [24] to 44.8% [30] to 66.1% [25] among COVID-positive patients. It is known to manifest early in the course of COVID-19 infection, and it has commonly persisted beyond the resolution of acute infection. In a study of 251 COVID-19 patients, 66.1% experienced myalgia at the time of diagnosis, and 25.4% of patients still experienced myalgia at the cessation of viral RNA shedding [25]. Many studies have concluded that myalgia occurs more frequently in women than in men and that women are significantly more likely to report it as a residual “long-COVID” symptom [27]. While the exact mechanism of myalgia has not been ascertained, the immune-mediated inflammation and cytokine reaction secondary to the SARS-CoV-2 attack on skeletal tissue ACE2 receptors has been widely accepted [26]. Another hypothesis suggests that viral damage triggers increased activity of lactate dehydrogenase and anaerobic glycolysis, thus increasing lactic acid levels, decreasing pH, and decreasing adenosine triphosphate (ATP) levels, ultimately leading to hypoxia-induced pain [28]. Regarding treatment, symptomatic management in the form of painkillers can be utilized to reduce myalgia in minor COVID-19 cases. In severe cases, there is an emphasis on ameliorating the hypoxia so the skeletal muscle can return to an aerobic state [28]. 

Headache

Headache is another common symptom of COVID-19 infection that presents early in the course of COVID-19 infection. In a study on 509 COVID-19 patients in Chicago, headaches were the second most common symptom after myalgia to present at onset with 37.7% experiencing headaches [30]. Another study of 450 COVID-19 patients conducted at Massachusetts General Hospital in Boston showed that significantly painful headaches impacted 26% of patients, and it further demonstrated that headaches were more prevalent in females, patients under 50, and Hispanic patients [71]. Studies have shown that in COVID-19, headaches typically present as new-onset and bilateral in the temporoparietal, occipital, and forehead region with a pressure-like pain [29]. Perhaps more interesting, however, is the number of studies that have underlined the prevalence of headaches in long-term COVID-19. Some have suggested that COVID-19 can cause New Daily Persistent Headache, which is unique from migraine or tension-type headaches and begins roughly two weeks post-recovery from respiratory symptoms [72]. There are several proposed pathogenic mechanisms for COVID-19-induced headaches. The first proposes that the headaches are a consequence of COVID-19-mediated hypoxia, ischemia, and systemic inflammation. A second suggests that ACE2 receptors on neuronal and glial cells are targeted by COVID-19, resulting in damage to these cells and consequent headaches. A third hypothesis proposes that the headaches are caused by the mass activation of T cells from COVID-19-induced cytokine storms [73]. Although there are no prevailing risk factors, it has been noted that headaches are a commonly presenting symptom in children [74]. As for treatment, tramadol has been proven effective against COVID-19-induced headaches [31]. Additionally, a recent study supports the potential use of calcitonin gene-related peptide (CGRP) antagonists for chronic headaches associated with COVID-19. This study further explains that CGRP receptors coexist with ACE2 receptors, the known targets of the COVID-19 virus, in the trigeminal ganglia, and thus may be responsible for COVID-19-induced headaches [75].

Dizziness

Another common neurological symptom of COVID-19 is dizziness. In a retrospective study of COVID-19 patients experiencing CNS symptoms, 16.8% reported dizziness [33]. General dizziness typically presents early in the progression of COVID-19, about five to six days post-infection [34]. Some studies have suggested that the presentation of dizziness alone can be an early indicator of COVID-19, exemplified by a case of a 78-year-old male who presented initially with only dizziness and unsteady walking before developing signs of respiratory distress several days later, at which time he was diagnosed with COVID-19 [32]. In a similar case, a 53-year-old, in December of 2019, presented to the hospital complaining of sudden dizziness for the past three days, for which she was treated with blood thinners and anti-cholesterol medications, with the doctors assuming a vascular origin. A COVID-19 differential was not considered until the fifth day post-admission [76]. The suspected MOA is the assault of ACE2 receptors in the capillary endothelium of neural tissue by COVID-19. Immune-mediated activity in response to COVID-19 infection may also contribute to dizziness [77]. Additionally, other studies have shown that COVID-19 dizziness can present with vertigo-like symptoms, possibly due to the primary COVID-19-induced inflammation of the macula and degeneration of otoliths [78]. Some studies have indicated that dizziness occurs more commonly in women [35]. Other studies have also found that dizziness presents significantly more commonly in Intensive Care Unit (ICU) patients compared to Non-intensive Care Unit (non-ICU) patients, with one study showing a dizziness prevalence of 22% versus 5% in ICU versus non-ICU patients, respectively [79]. No unique treatments have been discovered for this symptom.

Stroke

Stroke in COVID-19 patients, though less common than the above symptoms, can represent a serious risk to patients clinically presenting with endovascular-related comorbidities [36]. Stroke is a multifactorial disease state created by a variety of etiologies, which makes isolating the causes and subsequent mortality challenging. For this reason, analysis on a case-by-case basis is necessary to isolate how stroke may be evident in severe cases of COVID-19. Of the case reports available, all roads point to thromboembolism formation as the main perpetrator of ischemic stroke incidence. A systematic review and meta-analysis conducted by Zuin et al. revealed that the incidence of stroke post-recovery is higher compared to normal subjects from the general population, necessitating the need to understand the underlying pathophysiology of how ischemic events may occur in recovering COVID-19-afflicted patients [50,80]. A key feature in the progression of COVID-19 is the occurrence of thrombosis in the form of pulmonary emboli. In a case study written by Soliman et al. [51], a 69-year-old, unvaccinated female patient with severe COVID-19-related pneumonia developed a non-occlusive thrombus in the proximal middle cerebral artery, in addition to bilateral pulmonary emboli. Clinical presentation included a left facial droop and dysarthria, raising suspicion of a possible thrombotic event. The development of thrombosis is a rather logical incidence, given that patients with severe COVID-19 infections display all three elements of Virchow’s triad (hypercoagulability, immobility, and endothelial damage due to hyperinflammation). Hospital protocol dictates that patients at risk for thrombotic events be put on a prophylactic anticoagulation regimen using heparin and related anticoagulants. In this case, low molecular weight heparin (enoxaparin) was administered to reduce the risk of thrombosis. However, the patient’s labs showed elevated D-dimers and reduced kidney function, and the patient was switched to unfractionated heparin on hospital day 3. While the patient's respiratory condition was beginning to improve, D-dimer actually peaked and platelet levels continued to fall, indicating that the thrombocytopenia was not due to COVID-19, but perhaps heparin. On hospital day 8, the patient presented with left-sided facial droop, dysarthria, and mild left-sided upper extremity weakness. CT angiography showed a non-occlusive thrombus in the proximal right M2 branch of the middle cerebral artery. Intravenous tissue plasminogen activator (tPA) was administered, and the thrombus was evacuated. Apixaban was administered on discharge, and the patient was referred to hematology for follow-up [39]. This case presents an interesting paradox in the management of COVID-19 patients.

While thrombotic events in COVID-19 patients are not a common occurrence, physicians must be wary of heparin-induced thrombocytopenia, as it could lead to ischemic stroke in COVID-19 patients, as COVID-19 itself could also lead to thrombocytopenia. Physicians must also consider the cardiovascular and cerebrovascular risk factors in a given patient, as COVID-19 already creates a prothrombotic physiological environment. Those of older age and with predispositions may succumb to acute ischemic stroke [38]. Furthermore, according to a recent meta-analysis performed by Ferrone et al., patients with COVID-19 had a fourfold increased mortality if they had a stroke incident, indicating a significant need to manage stroke early in patients with COVID-19 [53,81].

Seizures

Although COVID-19 typically presents with the symptomatology of upper respiratory infections, seizures represent a neurological complication category that has been documented. Seizures fall under a large umbrella of clinical manifestations and only occur in 0.5% of COVID-19 patients [40]. There also seems to be a difference in manifestation depending on the strain and age population. Children hospitalized with COVID-19 from March to December 2020 had a 9% occurrence of seizures, but a 21% occurrence in January 2022, due to the Omicron variant [82]. For this review, we will be looking at acute symptomatic epileptic seizures and status epilepticus, as there have been a couple of cases documented in recent literature. One thing to note is that neurological complications often present themselves in a delayed capacity, rather than acute, and this is especially true in seizure manifestations [83].

Symptomatology of seizures is multivariate and depends on the location in the brain where it occurs, as well as on the type of seizure. A case report written by Dr. Asra Akbar and Dr. Sharjeel Ahmad at the University of Illinois College of Medicine examines the onset of six electroclinical acute onset focal seizures with impaired consciousness in a previously healthy seven-year-old girl who tested positive for COVID-19. Symptomatic presentation included loss of awareness with a duration of 10-15 seconds, along with episodes of “weak legs” over a couple of months. The patient had no previous history of seizures or any other neurological symptoms. Over time, the patient also experienced a loss of bladder control. Lab tests yielded normal levels, but the EEG revealed left-sided rhythmic low theta waves during behavior arrest, indicating epileptic activity. The patient was discharged on Keppra (levetiracetam), an anticonvulsant, which proved beneficial as the patient’s seizures were now controlled. In other cases, Ativan, phenytoin, phenobarbital, benzodiazepines, and Keppra were also utilized, depending on the type of seizure, ranging from generalized tonic-clonic to status epilepticus [40].

While the MOA of seizures is rather heterogeneous, there is evidence suggesting that viruses, in general, have been known to result in seizures. COVID-19 has been shown to bind to ACE2, which may be expressed in neuronal cells, leading to hyperinflammatory states in areas of the brain [84]. Furthermore, there has been research stipulating the neuroinvasive potential of the COVID-19 virus [85]. In addition, pro-inflammatory cytokine entry into the CNS may be a possible cause of epileptic activity [83].

Meningoencephalitis

Symptoms manifested by meningoencephalitis include a variety of presentations. It was found that, in a review of 61 cases of COVID-19-induced meningoencephalitis, the majority of individuals presented with seizures and altered mental status. Nuchal rigidity was observed in 9.25% of cases [42]. Another case, involving a young obese 41-year-old female, observed neck stiffness and photophobia along with headache, fever, and acute onset seizures. However, this patient presented without any respiratory failure [41]. In a case regarding a 68-year-old Brazilian man, the patient presented with clonic seizures, nausea, vomiting, and altered mental status, though Brudzinski, Kernig, and Lasegue signs were all negative [42]. The reason for the variety of symptomatic presentations is most correlated with the causative agent of meningoencephalitis.

Meningoencephalitis can be briefly defined as the inflammation of the meninges as well as the brain tissue itself. Agents that cause meningoencephalitis range from viruses to fungi, all of which cause a similar array of symptoms. However, the progression of the disease clinically varies from agent to agent. In the example of the 68-year-old Brazilian male, meningoencephalitis was caused directly by the COVID-19 virus itself, as shown by a negative CSF for *Streptococcus pneumoniae*, *Neisseria meningitidis*, *Haemophilus influenzae*, varicella-zoster virus, cytomegalovirus (CMV), and *Cryptococcus* spp. The quantitative polymerase chain reaction (qPCR) test revealed a positive result for COVID-19, and cranial MRI showed leptomeningeal enhancement as well as diffuse encephalitis. Previous research shows that the COVID-19 virus may exhibit neurotropic abilities, as it can penetrate the CNS and bind to neuronal cells with ACE2 receptors via a hematogenous route, causing hyperinflammatory cascades resulting in meningitis and encephalitis. Autopsy studies proved that viral particles were deeply embedded in frontal lobe neurons and endothelium [42]. While COVID-19 itself may cause meningoencephalitis via cytokine storm, there is another indirect pathogenesis that may also result in a similar clinical manifestation. Glucocorticoids, such as dexamethasone, are oftentimes used in patients with severe COVID-19 infections to lessen respiratory symptoms while protecting against organ damage and fibrosis. However, steroids have a rather potent immunosuppressive action, effectively shutting down the host’s immune response. While beneficial to a patient suffering from a COVID-19 cytokine storm, fungi such as *Cryptococcus* spp. and *Aspergillus* see this as a perfect opportunity. In a case regarding a 57-year-old male presenting with fever, chills, poor appetite, and symptoms of meningoencephalitis, steroid usage reactivated a dormant *Cryptococcus* infection, which was confirmed via lumbar puncture and India ink stain. Even with amphotericin B and oral flucytosine, the patient experienced six hypotensive episodes and eventually expired on day 42 of his hospital stay [44]. While glucocorticoids are a mainstay treatment in severe COVID-19 infections to protect against organ damage, a keen eye must be kept on opportunistic microbes and their reactivation.

Treatment for meningoencephalitis in COVID-19 patients depends on the causative agent. As there is an increasing amount of research indicating that the COVID-19 virus may contain neuroinvasive abilities, treatment for viral meningoencephalitis is of high importance. A case report written by El-Zein et al. [62] demonstrated the use of IVIg without glucocorticoids to avoid viral clearance delay. Five-day administration yielded a good response, and the patient’s stats returned to baseline, as well as his altered mental state. Caution must be used when administering IVIg, however, due to an increased risk of thromboembolism and subsequent stroke [45]. In cases of meningoencephalitis caused by extrinsic agents, such as fungi due to prolonged use of steroids, discontinuation of glucocorticoids and administration of antifungals is indicated.

Cerebellar Ataxia

Cerebellar ataxia is an uncommon complication of COVID-19, but it has been documented in multiple cases. Common presentations include dysarthria, dysmetria, bilateral dysdiadochokinesia, and truncal ataxia, along with broad-based gait and difficulty walking [46-53]. The timing of cerebellar ataxia onset following COVID-19 infection varies, but Chan et al. [64] indicated in their systematic review that the median onset was 13 days following initial COVID-19 symptoms, with a range of 0 to 48 days. It is worth noting that cerebellar ataxia symptoms represented the presenting symptoms of COVID-19 infection in some patients [47,51]. Due to the rarity of cerebellar ataxia as a COVID-19 complication, demographic risk factors are difficult to determine, but the male sex may represent a risk factor for COVID-19-induced cerebellar ataxia. In Chan et al.’s systematic review of COVID-19-induced cerebellar ataxia, 27 of the 33 patients they identified (81.8%) were males [64]. It is also notable that there have been pediatric reports of cerebellar ataxia, including a 13-year-old male [52] and a five-year-old male [50]. Treatment primarily consists of corticosteroid therapy, such as administration of high-dose IV methylprednisolone [46,48,52,53]. IVIG has also been added in some cases [49]. The elucidation of the MOA is still ongoing, but the causative mechanism is likely due to SARS-CoV-2’s direct viral neurotropism capabilities, as well as its ability to cross the blood-brain barrier and initiate cytokine storm in the host [86].

Acute Myelitis

While acute myelitis has been documented in patients infected with COVID-19, the incidence is rare, with roughly 1.4-4.6 cases per million [56]. The onset of symptoms induced by acute myelitis includes fever, headache, and, most importantly, muscle pain, which may be coupled with significant muscle weakness or progressive tingling [54,55]. The onset of symptoms typically ranges from one to two weeks following the initial COVID-19 infection [55,56]. However, one documented case in a 10-year-old male saw the onset of symptoms concurrently with the beginning of the COVID-19 infection [54]. Risk factors associated with acute myelitis are not well established, as the disease may generally present without any relevant comorbidities. However, it can be said that myelitis incidence may be highest in the second and fourth decades of life, typically secondary to an initial infection [55]. Standard treatment of acute myelitis consists of high-dose intravenous glucocorticoids, such as dexamethasone, for three to five days. Failure to respond to high-dose steroids would result in the use of plasmapheresis or immunomodulatory therapy (e.g., rituximab, cyclophosphamide). The MOA is unknown, but research shows that, due to the similarity in structure of the COVID-19 virus to other RNA viruses, CNS entry and neuroinvasiveness are plausible pathways. Entry through the blood-brain barrier or trans-synaptic transmission through the peripheral nervous system via ACE2 receptor binding is most possible [57].

GBS

Multiple cases of COVID-19-induced GBS have been documented [58-60,87]. The typical GBS symptoms are present in COVID-19-induced GBS, namely symmetrical ascending paralysis, paresthesia, and areflexia, which can progress to generalized tetraparesis [58,60,87]. However, it should be noted that COVID-19-induced GBS has been linked to worse outcomes than non-COVID-19-induced GBS [59]. The onset of GBS sequelae in relation to the onset of COVID-19 infection ranges dramatically. One study of 73 cases of COVID-19-induced GBS showed a range of GBS onset from eight days before to 33 days after COVID-19 symptoms [58]. Another study with GBS onset data for 165 patients obtained a range of GBS onset from 10 days before to 90 days after COVID-19 infection, with the vast majority of cases showing GBS onset after the onset of COVID-19 symptoms [59]. There appears to be a higher prevalence of post-COVID-19 GBS among males [58,59]. Treatment for these cases has been the standard for post-infection GBS-IVIg and/or plasmapheresis [59,60]. There are two main hypotheses regarding the mechanism behind COVID-19-induced GBS. The first hypothesis is that the same immune-mediated pathogenic mechanism that underlies non-COVID-19-induced GBS is to blame. In this theory, the infection generates an immune response that produces antibodies that cross-react with receptors at nerve membranes, resulting in nerve damage [58]. The second hypothesis suggests that direct viral neurotropism from COVID-19 is to blame for the severe neurological symptoms, rather than an immune-mediated injury [60].

MFS

Post-COVID MFS - a variant of GBS - has also been documented as a neurological complication of COVID-19 infection [58,59,61,62]. As with typical post-infection MFS, the typical post-COVID-19 MFS onset symptoms consist of ophthalmoplegia, ataxia, and areflexia, though dysphagia, dysphonia, diplopia, hypoesthesia, and back pain have also been reported [58-62]. Similar to post-COVID-19 GBS, these symptoms manifest days after the initial respiratory symptoms of COVID-19, ranging from 3 days to 20 days after the onset of COVID-19 [58]. Again, the male sex appears to serve as a risk factor [58,59]. Patients of advanced ages appear to progress more dramatically, with poorer outcomes than their younger counterparts [58]. Treatment for these cases has primarily consisted of IVIG [58-62]. The MOA, like COVID-19-induced GBS, is suspected to be either immune-mediated nerve injury or direct viral neurotropism [58,60].

PRES

PRES is a rare disease state characterized clinically by headache, altered mental status, visual disturbances, vomiting, seizures, and rapid, dramatic swings in blood pressure. On imaging, it is characterized by vasogenic edema in the posterior parieto-occipital regions of the brain [63]. Though uncommon, there have been multiple cases of COVID-19-related PRES. One study, which tracked 2,054 patients between March 4 and May 9 of 2020 in two New York City hospitals, found that 1.1% of the patients whose cases were severe enough to warrant neuroimaging were confirmed to have PRES (3 PRES cases out of 278 patients who received either CT or MRI imaging of the brain) [74]. Another case series, performed in a Multidisciplinary Pediatric Neurology Clinic associated with Seattle Children’s Hospital, included two patients who presented with PRES, confirmed by MRI imaging, following hospitalization with COVID-19 infection [88]. In addition, several individual case studies of COVID-related PRES have also been documented. Of these documented cases, the symptoms of PRES have manifested between 8 and 26 days following the onset of typical COVID-19 symptomology [63-66]. As with non-COVID-related PRES, risk factors include renal injury/failure, preeclampsia/eclampsia, autoimmune conditions, and immunosuppressive medications. The prognosis for PRES is good, with complete recovery reported in 75-90% of patients. Long-term neurological sequelae are a complication for a minority of patients, and mortality represents only 3-6% of PRES cases [89]. Treatment of PRES primarily consists of the management of symptoms and/or the removal of physiological triggers. For example, a patient who presents with PRES following an AKI will often see their PRES resolved once the AKI is adequately managed, and expeditious delivery of the infant in cases of pregnancy-related PRES serves to minimize PRES risks to the patient [89]. The pathophysiology of PRES, in general, has not been completely elucidated, but proposed mechanisms for COVID-19-related PRES include the disruption of the blood-brain barrier due to COVID-19-induced cytokine storm [66] and cerebral endothelial dysfunction induced by COVID-19 interactions with ACE2 receptors [64,65].

## Conclusions

Though the respiratory complications of COVID-19 are more front-of-mind, healthcare providers need to consider the possibility of neurological damage that their patients may face when infected with COVID-19 to provide adequate treatment. Furthermore, it is appropriate for providers to consider that neurological symptoms may be indicators of early COVID-19 infection, and this knowledge can be utilized to prepare an adequately swift care response for particularly vulnerable patients.

As the world continues to learn more about COVID-19 pathophysiology and clinical presentation, it will be essential to consider the impact that this infection can have on the human neurological system. Further studies are needed to provide more precise information regarding the incidence of COVID-19 neurological sequelae. Additionally, further longitudinal studies are needed and will be essential to the understanding of so-called “long COVID,” which certainly has some degree of neurological risk/involvement. Lastly, a confounding component of COVID-19 research that should be considered by the medical community is the possibility of varying neurological, respiratory, and mortality risks, dependent upon the specific strain of COVID-19 being studied. Data that differentiates between the different variants of COVID-19 is sorely lacking and may be a promising area of research moving forward.
